# Response: Commentary: Chronic PD-1 Checkpoint Blockade Does Not Affect Cognition or Promote Tau Clearance in a Tauopathy Mouse Model

**DOI:** 10.3389/fnagi.2020.00205

**Published:** 2020-07-02

**Authors:** Yan Lin, Leslie A. Sandusky-Beltran, Begona Gamallo-Lana, Adam Mar, Einar M. Sigurdsson

**Affiliations:** Department of Neuroscience and Physiology, Grossmann School of Medicine, Neuroscience Institute, New York University, New York, NY, United States

**Keywords:** tau, Alzheimer's disease, tauopathy, PD-1 blockade, antibody, therapy, mouse models, behavior

In a recent commentary on our article in Frontiers in Aging Neuroscience (Lin et al., 2020), Baruch and Yoles attempt to highlight perceived critical issues with our study and suggest that our scientific practice and conclusions are poor or misleading (Baruch and Yoles, 2020). They proclaim four main areas of concern—control groups, phenotype “shift,” presumptive motor impairment and antibody dosing regimen—which they argue obscure our conclusions. Here we respond to each area in turn in order to clarify the commenters' many misinterpretations and to encourage thoughtful and unbiased criticism of our and others' work.

The main purpose of our study was to assess treatment efficacy of PD-1 antibodies in tauopathy. We thus compared the phenotype of JNPL3 tauopathy mice randomized to anti-PD-1 antibody and IgG control groups. We agree that including age-matched wild-type mice would have provided a useful reference for the magnitude of any deficits or improvements. However, this would not change the clear fact that we observed no significant differences between anti-PD-1 antibody- and IgG control-treated JNPL3 groups in cognition or related tauopathy markers. With regard to a positive control group, as our main objective was not to show superiority of a particular treatment, concurrent testing of other reference compounds was beyond the scope of our study. Nonetheless, we did observe a significant behavioral effect of PD-1 blockade on a control measure—an increase in locomotor activity in an open field. This effect on locomotion may reveal an important caveat regarding therapeutic target specificity of PD-1 blockade (see below). Importantly, it further emphasizes our consistent lack of anti PD-1 antibody effects across a range of cognitive tests.

We indicated in our manuscript that the functional phenotype of our JNPL3 mouse colony currently appears considerably later than we originally described at earlier ages when we first started using this model many years ago (Asuni et al., 2007; Boutajangout et al., 2011). Such gradual shifts in phenotype have been observed in many transgenic Alzheimer's disease related mouse models with Aβ and/or tau pathologies in the over 20 years since their earliest description (Jankowsky and Zheng, 2017; Buck et al., 2018; Gotz et al., 2018; Hyman and Tanzi, 2019). With this in mind, and as we clearly outlined in the Discussion section of our article, we intentionally chose middle-aged animals to better relate to the prior work of Rosenzweig et al. (2019), starting the anti-PD-1 antibody treatment when the tauopathy mice have moderate to severe tau pathology. Indeed, the extent of tau pathology in our JNPL3 mice appears roughly comparable to their DM-hTAU model animals which, at their age treated, were described to “show pronounced cognitive deficits” (Rosenzweig et al., 2019). Although these models differ in tau mutations and background strains, experimental therapies should ideally be replicable across models (Latta-Mahieu, 2018). It is also notable that, in an earlier article, the authors emphasized that the therapy works at an advanced stage of pathology in Aβ plaque mice (Baruch et al., 2016). The commenters further pointed out that 6 of 22 mice died during the course of our study to argue that they were severely impaired. Deterioration and/or sudden death are rather common phenomena across transgenic Alzheimer's models, particularly with advancing age, and does not necessarily relate to motor deficits across the entire cohort. For example, although not clearly specified by the authors, a minimum of 12 of 67 animals were excluded from behavioral analysis due to severe motor impairments in the Rosenzweig et al. (2019) study (see page 4, difference in number of mice in Suppl Figure 3, *n* = 67 vs. Figure 2C, *n* = 55). No count of the number of excluded mice were provided in (Baruch et al., 2016).

Consistent with our observed phenotype shift, we categorically did not observe any gross or obvious motor impairments in our JNPL3 mice. We stated this observation explicitly in our article, and further confirmed and quantified locomotor activity using two sensorimotor behavioral tests as well as across five cognitive tests that require varying degrees of locomotion. The commenters highlight the fact that the mean distance traveled in the open field by the JNPL3 mice in our article (Lin et al., 2020) was approximately half of that observed in a prior publication (Boutajangout et al., 2011), and suggest that this indicates gross motor deficits. Later, they conversely argue that historical controls are meaningless in behavioral measures. What they neglect to mention are some of the key factors that likely contribute to this discrepancy, independent of motor impairments, including open field size and the age of the animals. It is well-known that mice tend to explore less both within a smaller arena (e.g., 56 cm diameter in the current vs. 70 cm diameter in the prior study) and at older ages (13–14 months in the current vs. 5–6 months in the prior study). For reference, a large-scale behavioral study of normal C57Bl/6J mice demonstrated an age-dependent reduction in locomotion within a 40 x 40 cm open field—with mean distance measures comparable and lower than we observed for JNPL3 in our testing conditions (Shoji et al., 2016; Lin et al., 2020). We have also previously found that other transgenic mouse models without motor impairments, such as htau mice at 11–12 months of age, travel 1,500–3,000 cm over an initial 15-min open field test (Congdon et al., 2016). Moreover, we transparently report the locomotor distances in our cognitive tests, such as the Barnes maze, where it is evident that JNPL3 mice typically travel further to reach the target and make more commission errors than wild-type reference controls, despite similar average velocities (Shoji et al., 2016). Finally, we also included fear conditioning in our cognitive test battery, a paradigm in which the expression of learning does not depend on locomotion, and found no significant impact of PD-1 blockade, consistent with our other cognitive assays.

Apart from the substantial evidence described above, it is unclear to us how anyone would argue that an 8 cm mouse traveling 30 m in 15 min (2 m/min) has a gross motor deficit that would preclude interpretation of our results. It is interesting to note that Baruch et al. (2016) and Rosenzweig et al. (2019) do not include any quantitative motor control tests in their studies despite acknowledging “animals that showed motor deficits were excluded from the behavioral analyses.” Moreover, cognitive performance on their primary cognitive task—a radial arm water maze—requires extreme motor capacity (e.g., swimming), yet no distance measures or trial omission errors are reported. Without proper motor controls or complete and transparent reporting of test results, their behavioral data are not easily interpretable, as enhancements in motor function might incorrectly be attributed to improvements in cognition. Given that our only significant effect—using the same anti PD-1 antibody as in their studies—was an increase in locomotor activity, a potential parsimonious explanation may be that PD-1 blockade acts primarily, or most effectively, to improve motor rather than cognitive function. We encourage future studies to include the appropriate motor controls and measures to avoid potential confusion in the field.

The commenters suggest that our experimental design, namely a weekly dosing schedule as opposed to the singular or intermittent dosing schedule previously reported (Baruch et al., 2016; Rosenzweig et al., 2019), may have somehow prevented us from observing a protection from cognitive impairment in our tauopathy mouse model. There is no evidence or scientific basis for such a claim. Various doses of anti PD-1 antibody were used in prior studies, with similar benefits shown with high vs. moderate antibody dose (Rosenzweig et al., 2019). The high dose, 1.5 mg/mouse, was also presented in the commenters' new data in Figure 1 (Baruch and Yoles, 2020; administered every 6 weeks). In addition, we noted that in the Baruch et al. (2016) study, the authors emphasized that “repeated treatment sessions are needed to maintain the beneficial effects on cognition and memory” and for “maintaining a long lasting beneficial effect on disease pathology” (Baruch et al., 2016). We thus specifically chose our dose to be 10 mg/kg, or about 1/5 of the aforementioned highest dose [assuming an average mouse weight of 30 g (1.5 mg/30 g = 50 mg/kg)]. Administering the dose per weight instead of the same dose for each animal should reduce variance in antibody levels between animals, whose weight typically varies by up to 20%. We did not find any information in previous articles regarding the half-life of this, or related anti PD-1 antibodies, but we are aware that the half-life of exogenous antibodies is on average about 2 weeks. Therefore, average circulating antibody levels are likely to be comparable in the Baruch et al. (2016) and Rosenzweig et al. (2019) studies and our report. Moreover, considering the typical half-life of exogenous antibodies, the short anti-PD-1 or anti-PD-L1 treatments used in these previous studies are likely to have resulted in continuous antibody exposure. Both higher doses administered at longer intervals and lower doses given at shorter intervals can lead to similar average circulating antibody levels. It is thus entirely unclear if the former approach has any particular benefits for treating neurodegenerative diseases.

In summary, we highlight the evidence, apparently overlooked or unappreciated by the commenters, showing that our experimental design was appropriate for testing our hypothesis, the age of our model was carefully selected based on tauopathy severity, and the mice had no gross motor deficits across multiple quantitative measures. Our dosing regimen was also chosen rationally, based on all existing information, and with no evidence that it is functionally distinct from earlier studies. There is absolutely no confusion that, in our model and experimental conditions, PD-1 checkpoint blockade does not significantly affect cognition or promote tau clearance. Hence, we stand by the title of the article. We wish to emphasize that we do not interpret our results as discrediting or invalidating any prior findings, but to add to existing scientific knowledge. We only suggest, as we did in our article, that further research in this area is warranted.

## Author Contributions

All authors listed have made a substantial, direct and intellectual contribution to the work, and approved it for publication.

## Conflict of Interest

ES is an inventor on patents on tau immunotherapies that are assigned to NYU. Some of these patents are licensed to H. Lundbeck A/S. None relate to PD-1 inhibition. The remaining authors declare that the research was conducted in the absence of any commercial or financial relationships that could be construed as a potential conflict of interest.
